# A Survey of Geosensor Networks: Advances in Dynamic Environmental Monitoring

**DOI:** 10.3390/s90705664

**Published:** 2009-07-15

**Authors:** Silvia Nittel

**Affiliations:** Spatial Information Science & Engineering, University of Maine / Orono, ME, USA; E-Mail: nittel@spatial.maine.edu; Tel.: +1-207-581-3681; Fax: +1-207-581-2270

**Keywords:** geosensor networks, wireless sensor network, environmental monitoring, sensor web, data management for geosensor networks, decentralized spatial computing, survey

## Abstract

In the recent decade, several technology trends have influenced the field of geosciences in significant ways. The first trend is the more readily available technology of ubiquitous wireless communication networks and progress in the development of low-power, short-range radio-based communication networks, the miniaturization of computing and storage platforms as well as the development of novel microsensors and sensor materials. All three trends have changed the type of dynamic environmental phenomena that can be detected, monitored and reacted to. Another important aspect is the real-time data delivery of novel platforms today. In this paper, I will survey the field of geosensor networks, and mainly focus on the technology of small-scale geosensor networks, example applications and their feasibility and lessons learnt as well as the current research questions posed by using this technology today. Furthermore, my objective is to investigate how this technology can be embedded in the current landscape of intelligent sensor platforms in the geosciences and identify its place and purpose.

## 1. Introduction

In the recent decade, several general technology trends have influenced the field of geosciences in significant ways. The first trend is the today more readily available technology of seemingly ubiquitous wireless communication networks, including access in remote and inaccessible areas without a wired communication infrastructure and often without even power lines. Furthermore, significant progress has been made in the development of low-power, short-range radio-based communication networks, which augment existing long-distance wireless communication networks. Second, the miniaturization of computing and storage platforms has led to low power consumption and has enabled novel computational platforms that can run on battery power for extended periods of time (e.g., several months with today’s technology). The third major trend is the development of novel sensors and sensor materials; this includes improved and size-reduced traditional sensors as well as the development of novel micro-scale sensors and sensor materials. For example, novel bio-chemical sensors can be used in the marine sciences or air pollution monitoring, or highly sensitive vibration and sound sensors have been applied for volcano monitoring, to name just a few. All three trends change the way of how events and phenomena in the environment are detected, monitored and reacted to. Overall, we can observe that platforms get more lightweight and portable, which opens up a plethora of new application areas for which platforms have been too expensive or too difficult to deploy before. Another important aspect is the real-time data delivery of many of the novel platforms that is possible today.

As a consequence, advanced platforms change the *paradigm* of how sensor data information is made available today. Adding more powerful, inexpensive computers to sensing devices of different scale transforms sensor stations from data loggers to intelligent, adaptive sensor platforms. The computational capability contributes to the ability of *onboard computing*; this includes local data analysis, data filtering and/or flexible sampling to adapt to events occurring and also the reduction of data transmission and battery consumption. Furthermore, via wireless networks an intelligent sensor platform can be connected to the internet and feed real-time data to remote applications. Overall, we can observe the trend that traditional sensor platforms have become more lightweight, portable, and intelligent and can deliver point-based data in real-time.

At the same time, a second technology development, which is still today mostly in a research stage, will add significant novel capabilities to modern geosciences. So-called g*eosensor networks* (GSN) are specialized applications of wireless sensor network (WSN) technology in geographic space that detect, monitor, and track environmental phenomena and processes [1–3]. Wireless sensor networks are a collection of tiny, untethered, battery-powered low-cost MEMS (micro-electro-mechanical systems) devices with limited on-board processing capabilities, storage and short-range wireless communication links based on radio technology, as well as sensing capabilities based on microsensors and sensor materials. Today, sensor nodes have the size of a cubic millimeter [4], and sensors can be at the size of a 1,000ths of a millimeter. Considering a remote sensing instrument as a ‘telescope’ to monitor environmental processes on the Earth, and a traditional sensor platform as an ‘eye’, a geosensor network can be viewed as an ‘*environmental microscope*’ providing a view to environmental processes at a spatio-temporal resolution of observations never available before. Leveraging this technology, we will be able, similar to microscopes elsewhere, to observe phenomena that were not or too difficult to measure before. Sensor networks also add the aspect of many-point based *regional* observations via the higher sensor node density in the area; thus, they deliver a more accurate estimation of the variations occurring in a spatial field. Similar to single-point intelligent sensor platforms, sensor network nodes perform local processing and filtering of sensed data, and at the same time collaborate with spatially neighboring nodes to detect interesting local ‘events’.

Both, the real-time aspect as well as the increased spatio-temporal resolution has brought new research challenges with regard to the approach of *modeling*, *monitoring* and *detecting* environmental processes. First, due to the real-time availability of sampling, data modeling and processing has a significantly shifted towards the monitoring and analysis of *dynamic* phenomena. This includes the observations of dynamic phenomena such as air pollution hot spots, or monitoring (groups) of mobile objects such as animals in a habitat. Second, it is necessary today to gain practical experience and experimentation of how to use this novel technology to detect and measure phenomena appropriately. This includes identifying the appropriate mix of hardware platforms for the phenomena type, the accessibility or inaccessibility of the observation area, hazardous environmental conditions, and power availability. For example, today wireless sensor network technology can be more effective for detecting and monitoring time-limited events (e.g., earthquake tremors) instead of continuous sampling in remote areas due to the battery constraints of geosensor platforms. With the much higher data rate and sample density, diverse practical problems exist such as accurately time-stamping samples. Third, new research challenges are posed in the computational field of spatial information science such as the development of algorithms for decentralized spatial computation, collaborative event processing and detection between collocated sensor notes, and lightweight, in-network data analysis. Last, but not least, novel intelligent sensor platform technology must be integrated with traditional and historic sensor data to augment data analysis and models. Another important aspect is the cross-domain and cross-platform availability of sensor data to leverage the deployment cost of sensor networks. Here, the ‘ideal’ is to create a web of real-time sensors that are accessible and sharable in a uniform way similar to data on the world-wide web today. For this, we need enabling standardized sensor service interfaces.

In this paper, I will focus mainly on the technology of small-scale geosensor networks, the novel research questions posed by deploying this technology, and show several applications today. Furthermore, my objective is to investigate how this technology can be embedded in the current landscape of intelligent sensor platforms in the geosciences and identify its place and purpose. The remainder of this paper is structured as follows: in Section 2, current technology developments are explored in more detail. Section 3 contains an overview of current geosensor network applications. Section 4 investigates newly posed research challenges in the field of spatial information science, and conclusions follow in Section 5.

## 2. Technology Developments

In this section, I will survey the technology, especially with regard to geosensor networks, in more detail.

### 2.1. Computing Platforms

Since the late 90s, via an DARPA-funded effort targeted research in the area of electrical and computer engineering has focused on the design of tiny computing platforms at the size of a penny [4], as well as the development of operating systems that are appropriate to run on and manage these small, resource-constrained platforms (e.g., TinyOS, Contiki and nesC) [5–7]. Since then, rapid advances in miniature, low-cost microelectronic and mechanical systems (MEMS) with limited on-board processing capabilities, storage and short-range wireless communication links have been made. The Universities of California at Berkeley and Los Angeles as well as MIT developed the first computing platforms that are commonly used in sensor network research projects today. The Intel/UC Berkeley Mica Mote series is commercially distributed today by Crossbow [8]. Other commercially available platforms are the TMote Sky, formerly distributed by Moteiv, which has now become Sentilla [9], a company co-founded by IBM, Texas Instruments and Sun Microsystems. The first Mica Motes did run on Texas Instruments processors and so both lines share some similarities. Table 1 provides an overview of the detailed specifications of currently available computing platforms. Ultimately, the objective is to develop truly miniature platforms at the size of sand grains, and create sensor networks consisting up of thousands or even millions of sensors sprinkled like “pixie dust” with microsensors at the size of a 1,000th of a millimeter, and set up collaboration in a self-organizing way and perform tasks in a decentralized way (“Smart Dust”) [4]. These efforts are today also commercially pursued by Dust Networks [10]. Combining ‘dust-sized motes’ with tiny sensor will provide an ‘environmental microscopic’ view to geophysical phenomena.

Computing platforms with such a significant reduction in size have required the rethinking and reimplementation of supporting software, too. One of the first software systems has been the development of an operating system, appropriately called TinyOS. This operating system has a very low memory footprint, i.e., a few kilobytes of code to store the entire operating system and a few hundred bytes of RAM to run it. It is available as open source software and further developed and extended in the community. Other operating systems and programming environments are Contiki [6] and Sentilla’s Perk Java-compliant platform for embedded 8-bit and 16-bit microcontrollers [9].

Besides designing small-footprint code, much research has been done to develop low-power, robust, ad-hoc communication *protocols* between sensor nodes. Since each node has a very limited reliable communication range (10–100 m), sending of messages in a sensor network is performed in a multi-hop way relaying the messages between sensor nodes until they arrive at their destination [11]. The protocols focus on how to route messages from a node to a destination using the least amount of energy and a robust topology, i.e., automatically adjust to temporary or permanent node or communication link failure.

Sensor networks have to be tasked, especially for intelligent sensor data collection. Since sensor networks are resource-constrained environments, data collection is integrated with message routing and coordination between nodes. This makes sensor networks cumbersome to program, debug and get to work reliably. A notable contribution to simple data collection programming interfaces is the technology of *sensor network database management systems (SDBMs)* [12]. SBDMS provide a declarative SQL-based interface so that a user can specify, which data he or she requests from the sensor network including the reporting intervals and simple aggregate processing.

Overall, the objective of software development is similar to software for today’s laptops, PCs and workstation: provide layers of software that can be reused for a large number of different applications, and need only software customization for specific applications, instead of integrated firmware packages. The reusability will bring the cost down, for both hardware and software.

The experience with small-scale geosensor networks in the field is still limited today, but the computing platforms and development software to task them have started to mature for real-world deployments. However, programming, deployment and testing are still cumbersome. Many applications today can be seen a exploratory prototypes, and the research domain is in an active state of exploring problems for which geosensor networks can be deployed to collect data and actuate the environment. Many applications use the inexpensive, open-source Mica Motes platforms, i.e., the sensor nodes are still rather match-boxed sized rather than sub-millimeter and use AA batteries for power. The general idea of geosensor networks is to deploy sensor nodes without wires, i.e., without power line and communication lines. Such untethered geosensor networks are easier to deploy in remote areas, but have the disadvantage that the energy supply is limited, and thus, they are currently better used for detecting and monitoring short-term events such as volcano tremors. For areas closer to a power infrastructure, wired sensor nodes are more reliable and less constrained and are more suitable for continuous monitoring.

### 2.2. Sensors and Microsensors

Similar to the MEMS production to create tiny computers, new sensors and sensor materials are under development today, made possible by modified semiconductor fabrication technologies. These processes include deposition, photolithography, etching and wet etching, and others. MEMS sensors are made up of components between 1 to 100 micrometers in size (i.e., 0.001 to 0.1 mm). They are made out of silicon, polymers or metals such as gold, titanium, or, platinum. The microsensors use standard interfaces to attach to MEMS computing devices.

For geosciences, sensors such as temperature, humidity, light, acoustic or vibration sensors are commercially available today. Of particular interest will be micro-chemosensors that can detect very small concentrations of certain gases in the air. For example, SAW (Surface Acoustic Wave) chemosensor are used to analyze and detect gas mixes such as halogenated volatile organic compounds (e.g., chlorine, fluorine), which are used in solvents and herbicides. Furthermore, bio-chemical microsensors can detect small concentrations of spores, or bacterial growth in small spaces. The development of new sensor materials will truly make sensor networks ‘environmental microscopes’ of unknown proportions. This research field is very active, and we can expect that first prototypes of bio-chemical microsensor boards for geosensor networks will be commercially available.

Despite the availability of tiny sized sensor platforms, they do not replace existing larger scale sensor platforms for the geosciences. Instead, the variety of sensor platforms will scale from tiny to match-boxed sized as well as medium sized to in-situ large instruments to remote sensing devices. The appropriate platform is defined by the phenomenon of interest to observe, and different, sometimes concurrently deployed platforms of different scale need to be explored.

## 3. Geosensor Networks Applications

Following, I will give an overview of examples in different application domains today. I classify three application types that can be distinguished based on their observation characteristics. The first class encompasses terrestrial ecology observation systems; in these applications *continuous monitoring* is typical to e.g., assess plant health and growth circumstances or to observe and measure geophysical processes. The second application class is characterized by real-time *event detection*; as example I use a volcano sensor network deployment to elucidate types of event monitoring and data processing challenges. The third class of applications includes *mobile sensor nodes* such as found in aquatic observation systems using drifter networks or animal tracking.

### 3.1. Terrestrial Ecology Observing Systems

*Agricultural Sensing System, Australia*: An agricultural wireless sensor network project was started in Australia in 2006, and the study has been completed in 2008 [13]. The area tested was a nectarine orchard covered with around 270 sensors using Crossbow's Motes, and a gateway connected it to the Internet. The data was collected with regard to air temperature, relative humidity, location (GPS), ambient light, solar radiation, barometric pressure, precipitation, wind speed, wind direction, leaf witness as well as soil moisture sensors. Both the measuring of soil moisture variability as well as information on tree canopy helps increasing the productivity of the orchard, i.e., increase it fruit yield and optimize irrigation usage. The WSN collected soil moisture measurements at three soil depths, at up to 100 locations, each hour for the duration of the study. Soil moisture as well as weather information and irrigation uses determined the ‘health’ of the orchard; daily network health statistic alerts were sent via SMS to a mobile phone.

*Networked Soil CO_2_ Sensing Systems, UCLA*: The objective of this CENS-related project at UCLA has been to examine the spatial and temporal heterogeneity of a soil environment within a forest area in the James Reserve. The soil environmental measurements are collected with ten stations, each of which consists of an array of belowground sensors including soil CO_2_, soil temperature, soil water content, and aboveground air temperature, relative humidity, and photosynthetic active radiation. Models are used that relate the aboveground microclimate and the soil measurements to belowground measurements made by the project’s sensors to ‘map’ the microclimate in a fine-grained resolution, and investigate soil CO_2_ fluxes depending on the local characteristics of the forest cover story [14].

### 3.2. Geological Observation Systems

*Volcano observation, Harvard, New Hampshire and North Carolina*: Research groups from Harvard, University of New Hampshire and North Carolina have collaborated for several sensor network deployments in the remote, inaccessible area at the active volcano Reventador in Ecuador in 2005–2008 [15–17]. The objective of the sensor network was to test the ability to detect and measure tremor events of the volcano; the deployment period varied around three weeks. Typically, the geosensor network consisted of 16 TMotes Sky using seismo-acoustic sensors; it was deployed over a linear stretch of 3Km, pointing away from the volcano. The sensor nodes used short-range, battery-preserving wireless multi-hop communication to communicate with each other and relay data, and the sensor network was connected via a long-distance radio communication link to a Freewave radio modem at a make-shift observatory. The observatory consisted of a laptop acting as a coordinator and storing the sensed data, and a Freewave radio modem, which was powered a solar-panel powered car battery.

The goal of the sensor network deployments was to detect and measure tremor events. First, those events needed to be detected, since monitoring continuously would deplete the batteries fast. To do so, the nodes were programmed to compare a short-term average with a long-term average based on locally stored samples. If the difference was bigger than a threshold, a node would send a message to the base station. If a sufficient number of nodes reported an event, the base station triggered a data collection request to all nodes in the sensor network. Based on the event message, data was collected at high frequency, i.e., seismic signals at ca. 100 Hz. The data was cached locally on a flash drive, and then relayed to the base station. Since the wireless communication bandwidth is low (ca. 10 Kbyte/s of real data without message header overheads), it took up to 1 hour until all data was transmitted to the base station. Before a triggering data collection event, nodes used the local storage as a ring buffer comparing the short-term and long-term averages.

### 3.3. Aquatic Observing Systems

*Mobile* geosensor networks consist of individual sensor nodes that are mobile or attached to mobile objects such as cars [18], animals [19] or ocean buoys [20]. Applications for mobile geosensor networks are tsunami early warning systems [21], or coastal and ocean observations conducting contamination detection [20]. In the NAMOS project at UCLA, a ‘hybrid’ sensor network system was constructed, which consists of a wireless stationary buoy sensing system and a mobile robotic surface vehicle capable of sensing and sampling. This sensor network was deployed in Lake Fulmor on the James Reserve to obtain both high-resolution temporal information of environmental parameters (provided by the stationary buoys) and data from specific locations using the capabilities of the robotic boat to study plankton dynamics. Environmental and event information collected from the buoys was used to guide the robotic boat.

## 4. Research Challenges

Small-scale geosensor networks pose a plethora of new research challenges. Beside the objectives to develop smaller computing nodes, novel renewable battery supply as well as new microsensors, many new research problems are posed at the software level as well as getting more experience with robust deployment, testing and data analysis.

This section investigates four areas of research challenges related to geosensor network: *first*, programming geosensor networks is cumbersome and complex today; it requires in-depth technology and programming expertise, however, user-friendly applications programming interfaces (APIs) are needed, which can easily be used by domain scientists to experiment with such platforms. *Second*, to reduce energy consumption and extend the application lifetime of geosensor networks, novel algorithms have to be developed that detect, monitor and track environmental phenomena ‘*in-the-network’* using spatially localized computation at the phenomenon’s location instead of pulling all data from the geosensor network and performing traditional data analysis in a centralized geographic information system (GIS). *Third*, to process both geosensor network data as well as traditional gesosensor data in real-time, a sensor data stream paradigm needs to be used for data management. *Fourth*, with continuously wider spread use of geosensor platforms, sensor data integration is of key importance to enable a so-called “Sensor Web” making it easy to share one’s sensor data streams as well as leverage the real-time sensor data from other deployments for one’s applications.

Two of these challenges are specific to geosensor networks compared to generic wireless sensor networks: the field of spatial information science has accumulated a huge knowledge of computational spatial data analysis methods over the past 40 years. However, these models of space and phenomena and accompanying algorithms are tailored towards sparse sensor deployments and powerful computers. The main challenge is to apply this domain knowledge conceptually to the new scale of sensors and phenomena and redefine algorithms so that they can be run in a lightweight, energy-efficient, decentralized fashion within a geosensor network. Second, geosensor network technology is only the latest addition to a landscape of widely deployed larger sensor platforms that range from remote sensing to ocean buoys and weather stations. Being able to integrate the sensor data at different temporal and spatial scale seamlessly as well as using e.g., small-scale geosensor network event detection such as the detection of volcano tremors to trigger sensing of remote sensing platforms and vice versa is significant challenges and opportunities today.

### 4.1. Application Programming Interfaces for Geosensor Networks

Due to the constrained computing, storage, communication and energy resources of wireless sensor networks, the communication protocol stack is, like many other aspects of the operating system for such systems, significantly collapsed and instead of the typical seven ISO/OSI layers the stack is reduced. Therefore, routing is tightly integrated with the data collection layer such that routing communication messages and data collection tasks have to go hand-in-hand. Developing such ‘data-centric’ routing and data collection programs [22] and optimizing them with regard to minimizing energy consumption as well as robustness against link and node failure is a challenging task. It requires significant programming expertise. Today, programming interfaces are C or Java-based. The programming language nesC [7] was developed for such constrained sensor networks; the TOSSIM [23] and Contiki [6] programming environments combine code development with simulation so that debugged and simulated code can be installed and tested on sensor nodes.

To task sensor networks for applications today, in-depth operating systems and programming knowledge is required. This expertise, however, is often not the foray of the intended users of sensor networks, mostly scientists today. Scientists do understand the environmental processes that they are interested in observing and monitoring; they need to be able to easily re-task a sensor network to explore the deployment with regard to different sampling settings and spatial layout. Therefore, data collection and re-tasking have to be simplified significantly so that users can be more autonomous from sensor network programmers. To achieve this goal the database community has proposed (and implemented) a SQL-based database interface approach [12]. Using an SQL interface, data collection tasks are formulated as declarative spatio-temporal queries such as “SELECT vibration FROM sensors WHERE vibration > threshold SAMPLING EPOCH 1h”. SQL-based queries are simple to formulate, and the query execution and optimization are, similar to traditional DBMS, automatically generated by the DBMS software system. Thus, the domain scientist is relieved from developing, testing and optimizing programming code, and can use a programming interface that allows him/her to define the necessary tasks in a user-friendly way. The optimization and ultimately self-adaptive execution of the data collection task are delegated to the DBMS run-time system without the user having to worry about the details. TinyDB is the first open-source ‘sensor database management system’ available today [12].

Nevertheless, the DBMS internal code for sensor networks is significantly different from traditional DBMS. First, the memory foot-print of the DBMS is very small (a few Kbyte) as opposed to millions of lines of code of commercial DBMS. Second, few data items are stored, but queries are used to task the sensor network and *acquire* sensed data in the user-specified intervals, while in traditional DBMS all queries are executed on large amounts of stored and indexed data [12]. Third, query execution has to be combined with query routing and data collection topology in a fundamental way, so that appropriate query plans can be generated automatically. Fourth, although not computationally complete as a programming language SQL has the ability to define aggregate function such as min, max, average or others. These functions are also available for sensor network DBMS interfaces. The internal approach to computing aggregates has to be combined with routing and data collection, too.

### 4.2. Decentralized and Collaborative Spatial Computation

Due to the scale and limitations of wireless geosensor networks, the data processing paradigm changed fundamentally as mentioned before. Overall, the battery limitations and the objective of maximizing the application lifetime are driving the paradigm to collect data and detect events. The biggest energy sink is the use of the wireless radio communication, since sending data consumes about 800 times more energy than computing the same amount of data on the local chip [12]. Thus, design of algorithms is driven by the objective to minimize communication, i.e., the *number* of messages to be sent between nodes, and the *size* of the messages. Using a wireless sensor network purely as a raw data collector depletes batteries fast, because the data is forwarded in a multi-hop fashion between nodes to the network’s base station. Each node in the network attaches its own raw data, and the message size increases with each network ‘hop’. Thus, nodes close to the base station energy-deplete significantly faster compared to leaf nodes in the network.

The general idea of the computational paradigm shift is to push the computation into the ‘network’. Instead of forwarding large amounts of raw data to be analyzed outside of the sensor network, local processing and storage can be utilized to only forward ‘interesting’ data (e.g., data above or below a certain threshold) or events (e.g., a tremor of a volcano). Also, neighboring nodes collaborate on detecting ‘interesting’ data, collaboratively filter data, or detect local events (such as the boundary of a toxic plume and its motion). This is advantageous since events such as a contamination event is locally confined to one or several regions, and can be computed by the nodes deployed within the confines of the event, while distant nodes are irrelevant for the processing. Thus, the locality of events can be leveraged by local in-network computation and collaboration, which at the same time reduces communication since only spatially neighboring nodes exchange messages, and only local decision making is necessary.

Typical types of spatio-temporal queries over geosensor network concern either continuous phenomena such as microclimates or event detection such as toxic plumes or wildfires. Many environmental phenomena, such as a microclimate within an orchard or a gas concentration in an open space are *continuous*. Sensor data samples, however, are discrete and point-based, and the resolution is based on the density of sensor nodes in the observation region. The challenge is to provide an accurate and precise estimation of the dynamic spatial field based on limited discrete point samples collected by the sensor nodes. Well-know data analysis and estimation techniques need to be redesigned to be lightweight and processed collaboratively ‘in the network’ while minimizing communication [24,25].

Instead of estimating dynamic spatial fields quantitatively, for queries targeting to identify *events* such as detection of a toxic plume or the occurrence of a flood, finding the boundary of such a phenomenon can be sufficient. The boundary indicates the (dangerous) region of the event, and the user typically knows how to define the event (e.g., a chemical gas concentration above a certain threshold). Identifying only the boundaries and tracking their changes over time can save energy significantly since processing is localized to the boundary, and can be minimized or suppressed elsewhere in the network.

Much work has been accomplished in the area of in-network boundary detection, and monitoring. Part of the work is focused on identifying *boundary points* [26]; other work focuses on computing the *geometry* of the phenomena in the network [27], and the third part of the work tracks *changes of the object boundaries* in the network [28–30]. The third aspect can be classified into approaches that a) identify the topology of the object boundary and track its topological changes (e.g., “does the toxic plume split?” “Do the Witch fire and Poway fire merge?”) [28,29], and b) approaches that track the geometric changes of the boundary incrementally in the network [30]. It is foreseeable that with the availability of more in-network algorithms of well-known spatial data analysis methods, geographic information systems (GIS) will disappear as a centralized data analysis tool for raw sensor data. Now, the “sensor network is the GIS” [31]. Another approach related to processing raw, noisy and sometimes missing sensor network data is to use model-based collection methods [32,33]. Here, the user can define confidence values necessary for requested sensor data, and the in-network data acquisition algorithm takes those guarantees into account, i.e., automatically correlates data spatially or increases local sampling frequency.

Several technical challenges with regard to the time synchronization of data sampling in sensor networks has been elucidated in the volcano deployment application. Here, the sensor network collected a rapid time series of data during a tremor; each sensor node produced a stream of data tuples including a location and time stamp. Once collected at the base station, the data tuples need to be sorted accurately to compute an event correctly. This is challenging since it is difficult to synchronize the local clock of all sensor nodes, which, however, is a vital aspect in this specific scenario. The second challenge is to be able to catch ‘significant’ triggering events to start a data collection, and e.g., not to catch a precursor event and miss the significant event occurring right afterwards. Thus, domain knowledge about the nature of volcano tremors needs to influence the programming.

### 4.3. Real-Time Sensor Data Streams

Seeing small-scale geosensor networks as a component of a new generation of inexpensive, smaller, mobile and intelligent sensor platforms, we can abstract the ‘output’ of a sensor network as a data *streams* of sensor information with regard to the region covered by the sensor network. On the other hand, the availability of sensor platforms of different size provides huge collections of concurrent, geo-referenced sensor data streams in *real-time*. The challenge is to build appropriate data management technology to query, process, mine and analyze the data streams in *real-time* to find trends and identify events. In database research, data stream management systems have been developed in the last decade. The basic technology of rapidly processing large numbers of incoming data streams is appropriate for real-time sensor data streams. However, today data stream engines are focused on business applications such as monitoring credit card or stock market transactions with simple structured data tuples, and geo-referenced sensor data streams are not supported well yet. Extensions to the temporal, but not spatial data models, and the query languages and operators are necessary to enable support for rapid processing of sensor data streams as well as their integration and analysis using powerful computer clusters.

### 4.4. Sensor Web

With an increasing number of real-time sensor data streams available online via the internet, users are interested in a platform that enables sharing and finding such sensor data streams as well as easily writing new applications using this data. Such an environment is described as the ‘sensor web’. It needs to allow users to select sensor data streams of interest depending on location, resolution, reliability and sensor type, and easily build applications for sensor data integration, mapping, analyzing in real-time and archiving. On the other hand, similar to the WWW, users should be allowed to register their own sensor resources within such a sharing environment. Notably, scalability issues to manage and share such large amounts of sensor data are important.

Microsoft’s SenseWeb [34] is a peer-based software that allows user to run the software locally to make local sensor data available, and on the other hand mashes up sensor data delivered via SenseWeb to a map interface. It provides interactive tools to pose queries to the sensors and visualize data, along with authentication-based access to manage sensors. The interface resembles functionality typically available via a GIS. For example, users can visualize individual attributes of a sensor data stream, which are displayed on so-called layers. For example, a layer could show the distribution of measured CO_2_ over a city region. Using raw sensor data, contour maps can be computed to visualize the spatial distribution and variation of the sensed data. Layers can be overlaid, clipped, zoomed in, etc. to perform visual data analysis.

The *SenseWeb* application indicates that sensor data stream sharing and integration will be vital in the future to leverage deployment cost, and *SenseWeb* is a step in this direction. However, for large scale national and international sensor platform data integration and interoperability, cross-platform open standards have been proven to serve as the enabling technology in the past, and likely will be the case with real-time sensor data streams. *Standardization of interfaces* to connect to and access sensor devices as well as standardized sensor data representation using technology like XML or others are necessary to make such systems interoperable. Providing standardized interfaces and open-source software will make access to and sharing of sensor data uniform, so that scientists can share, find, combine, and query real-time sensors in geographic regions around the globe. The OpenGIS Consortium has been conducting such standardization of sensor platform interfaces for several years, and several standardization are available today [35].

## 4. Conclusions and Outlook

Today, the domain of geosciences is at the brink of a new wave of technology: ubiquitous wireless communication networks including long and short-range communication technology, intelligent sensor platforms including localized and collaborative processing as well as untethered deployment of sensors using battery power. This leads to a fundamental paradigm shift of how we can sense, monitor and track dynamic phenomena in real-time in the environment. The technology of small-scale geosensor networks is still in its infancy today. In this paper, I gave an overview of the current technology and the expected future developments, described a selection of applications and some lessons learnt, and explored the current research questions posed by this technology today.

## Figures and Tables

**Figure 1.**
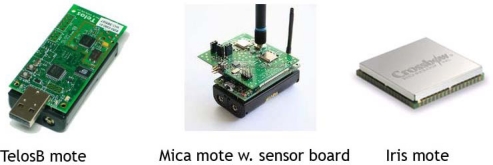
Computing platform overview.

**Figure 2.**
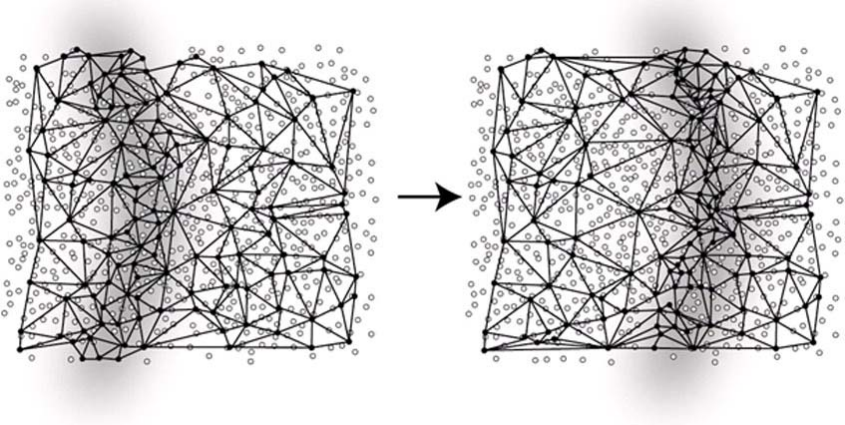
Tracking continuous phenomena over space and time using geosensor networks.

**Table 1.** Technical specification of computing platforms.

**MODEL**	**MICAz**	**IRIS**	**IMote 2**	**TelosB**	**Tmote Sky**
Processor	8 MHz MPR2400	8 MHz XM2110CA	13–416 MHz PXA271	8 MHz MSP430	8 MHz MSP430
OS	Moteworks	Moteworks	TinyOS, Linux	TinyOS	TinyOS
Memory	4 Kbytes	8 Kbyte	256 Kb SRAM	10 Kbyte	10 Kbyte
Flash	128 kB	128 kB	32 MB	48 kB	48 kB
Storage	512 kB	512 kB	32 MB SDRAM	1 MB	1 MB
**COMMUNICATION**					
Radio	802.15.4	802.15.4	802.15.4	802.15.4/ZigBee	802.15.4
Data Rate	250 kbps	250 kbps	250 kbps	250 kbps	250 kbps
indoor	20–30 m	>50 m	30 m	20–30 m	50 m
outdoor	75–100 m	>300 m		75–100 m	250 m
**ENERGY**					
Transmit current	11–17.4 mA	10–17 mA	44/66 mA		21.8 mA
Receive current	19.7 mA	16 mA	44/66 mA	23 mA	19.5 mA
Active current	8 mA	8 mA	31 mA	1.8 mA	500 μA
Sleep current	<15 μA	8 μA	390 μA	1 μA	2.6 μA
**SIZE**					
Size (mm)	65 × 31 × 6	65 × 31 × 6	36 × 48 × 9	65 × 31 × 6	65 × 31 × 6
Weight (g)	18	18	12	23	23
